# A novel approach to alleviate acetaminophen-induced hepatotoxicity with hybrid balloon flower root-derived exosome-like nanoparticles (BDEs) with silymarin via inhibition of hepatocyte MAPK pathway and apoptosis

**DOI:** 10.1186/s12964-024-01700-z

**Published:** 2024-06-18

**Authors:** Jisu Kim, Chao Gao, Pengcheng Guo, Jianyong Sheng, Jianxin Wang

**Affiliations:** 1 Department of Pharmaceutics, School of Pharmacy, Fudan University and Key Laboratory of Smart Drug Delivery, Ministry of Education, Shanghai, 201203 People’s Republic of China; 2 Science and Technology Innovation Center, Guangzhou University of Chinese Medicine, Guangzhou, 510405 China; 3 Institutes of Integrative Medicine, Fudan University, Shanghai, 201203 People’s Republic of China

**Keywords:** Plant-derived exosome-like nanoparticles, Balloon flower root-derived exosome-like nanoparticles, Hybrid exosomes, N-acetyl-ρ-aminophen-induced liver injury, Drug delivery systems

## Abstract

**Introduction:**

Balloon flower root-derived exosome-like nanoparticles (BDEs) have recently been proposed as physiologically active molecules with no cytotoxicity. However, the therapeutic effects of drug-induced hepatotoxicity of BDEs have not been elucidated. BDEs contain a large amount of platycodin D, which is widely known to be effective in regulating inflammation and ameliorating systemic toxicity. Thus, the main therapeutic activity of BDEs is attributed to inhibiting the inflammatory response and alleviating toxicity. In this study, we fabricated the hybrid BDEs fused with liposomes containing silymarin (SM) to enhance the synergistic effect on inhibition of acetaminophen-induced hepatotoxicity (APAP).

**Objective:**

Considering the potential therapeutic effects of BDEs, and the potential to achieve synergistic effects to improve therapeutic outcomes, we constructed hybrid BDEs with a soy lecithin-based liposome loaded with SM. Since liposomes can provide higher thermal stability and have greater structural integrity, these might be more resistant to clearance and enzymatic degradation of drug molecules.

**Methods:**

Hybrid BDEs with liposome-loaded SM (BDEs@lipo-SM) were fabricated by thin-film hydration and extrusion. BDEs@lipo-SM were characterized using dynamic light scattering and high-performance liquid chromatography. After confirmation of the physical properties of BDEs@lipo-SM, various therapeutic properties were evaluated.

**Results:**

BDEs@lipo-SM were internalized by hepatocytes and immune cells and significantly decreased mRNA expression of apoptosis and inflammation-relevant cytokines by inhibiting the hepatocyte MAPK pathway. BDEs@lipo-SM significantly induced an increase in glutathione levels and inhibited APAP-induced hepatotoxicity.

**Conclusion:**

From this study, we know that BDEs are reliable and safe nanovesicles containing natural metabolites derived from balloon flower, and they can facilitate intercellular communication. BDEs are also easily modified to enhance drug loading capacity, targeting effects, and long-term accumulation in vivo. BDEs@lipo-SM have therapeutic benefits for acute liver injury and can alleviate cell death and toxicity. They can be efficiently delivered to the liver and effectively inhibit APAP-induced hepatotoxicity by inhibiting the MAPK signaling pathway and apoptosis, which accelerates liver recovery in the APAP-induced acute liver injury model. These findings highlight that BDEs represent an attractive delivery vehicle for drug delivery.

**Graphical abstract:**

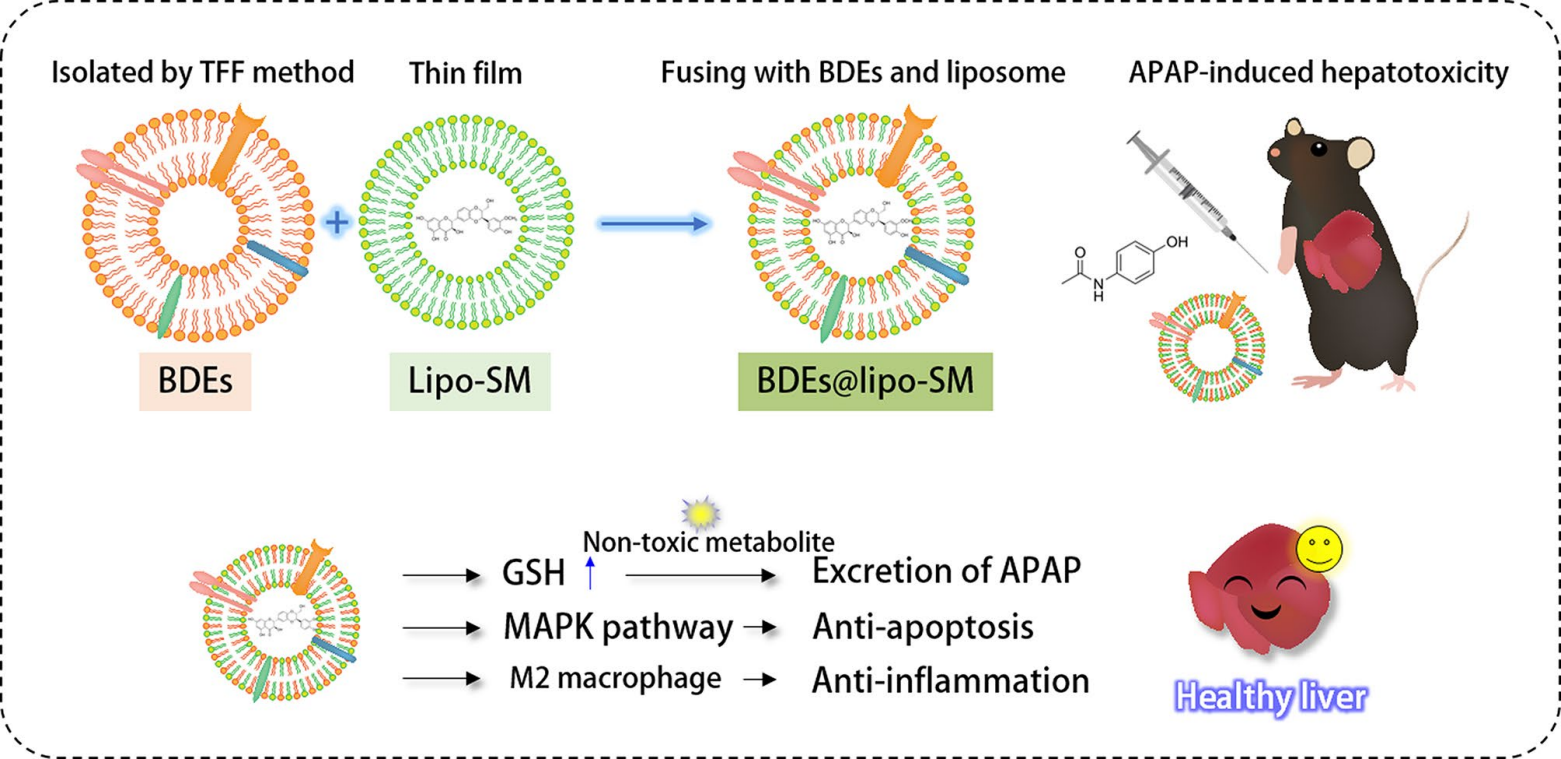

**Supplementary Information:**

The online version contains supplementary material available at 10.1186/s12964-024-01700-z.

## Introduction

Acetaminophen (N-acetyl-ρ-aminophen, APAP) is the most popular analgesic and antipyretic drug and is widely used for mild to moderate pain and fever worldwide. APAP is considered safe to use at the recommended doses, but it can induce severe liver toxicity, even death, following intentional or non-intentional overdose. In the United States, drug-induced liver injury (DILI), including APAP-induced hepatotoxicity, is the main cause of acute liver failure, which suddenly affects healthy individuals and requires the urgent emergency liver transplantation [1, 2]. Optimal management of DILI resulting from APAP toxicity remains to be elucidated and is associated with adverse patient outcomes with increased morbidity. Thus, the development of a novel approach to the treatment of DILI is urgently needed.

In 1963, the US Food and Drug Administration (FDA) approved N-acetylcysteine (NAC) as the main treatment agent for APAP overdose [3, 4]. APAP hepatotoxicity occurs through the formation of the toxic N-acetyl-ρ-benzoquinone imine (NAPQI) metabolite, resulting in glutathione (GSH) depletion and the formation of APAP protein adducts [5]. Adduct formation on mitochondrial proteins modulates respiratory chain function, producing elevated levels of free radicals such as superoxide, which promotes extensive cell death. NAC, a GSH precursor, is used as an antidote to APAP overdose by providing cysteine for glutathione synthesis and can act to detoxify oxidizing radicals of APAP. Unfortunately, therapy with NAC comes with limitations such as a need of high doses and long treatment times due to its poor bioavailability. Furthermore, cases of nausea, vomiting, diarrhea, and anaphylactoid reactions have been reported. Thus, a novel treatment approach to the treatment of DILI is required without such limitations and severe side effects [6, 7].

In Chinese medicine, balloon flower root has been traditionally used for the treatment of bronchitis, asthma, hypertension, allergies, and other inflammatory diseases due to its beneficial bioactive properties [8–11]. Balloon flower root contains saponin, specifically platycodin D, as the main active component that can potentially inhibit inflammatory pathways, promote AMPK expression and protect from oxidative stress damage. Recently, the protective effects of balloon flower root and its bioactive molecules on acute liver toxicity have been reported [12]. According to a previous report, platycodin D may partially inhibit hepatocyte apoptosis by inactivating the MAPK signaling pathway, while reducing mitochondrial oxidative stress in vivo [10, 13, 14]. However, it is not warranted to investigate therapeutic doses of the original raw form balloon flower root for its clinical benefits and bioavailability of phytochemical and bioactive molecules in the circulation [15].

Herein, to improve therapeutic outcomes through enhanced bioavailability and to achieve a better therapeutic effect on drug-induced liver injury, we successfully isolated BDEs at high yield using a tangential flow filtration (TFF) method and demonstrated the development of a BDE-based hybrid strategy and discussed the therapeutic potential of the formulation in treating APAP-induced hepatotoxicity [16–18]. In addition, hybrid BDEs were prepared with improved stability, showing significantly enhanced accumulation in the liver and prolonged circulation compared to the free BDE liposome. In conclusion, BDEs can be a promising candidate as a drug delivery system for liver injury therapy and the combination of BDEs with SPC-based liposome-loaded SM (BDEs@lipo-SM) can be exploited to deliver therapeutic cargoes to cells and to provide improved drug protection during delivery without hepatotoxicity (See Schematic illustration).

**Scheme 1:**
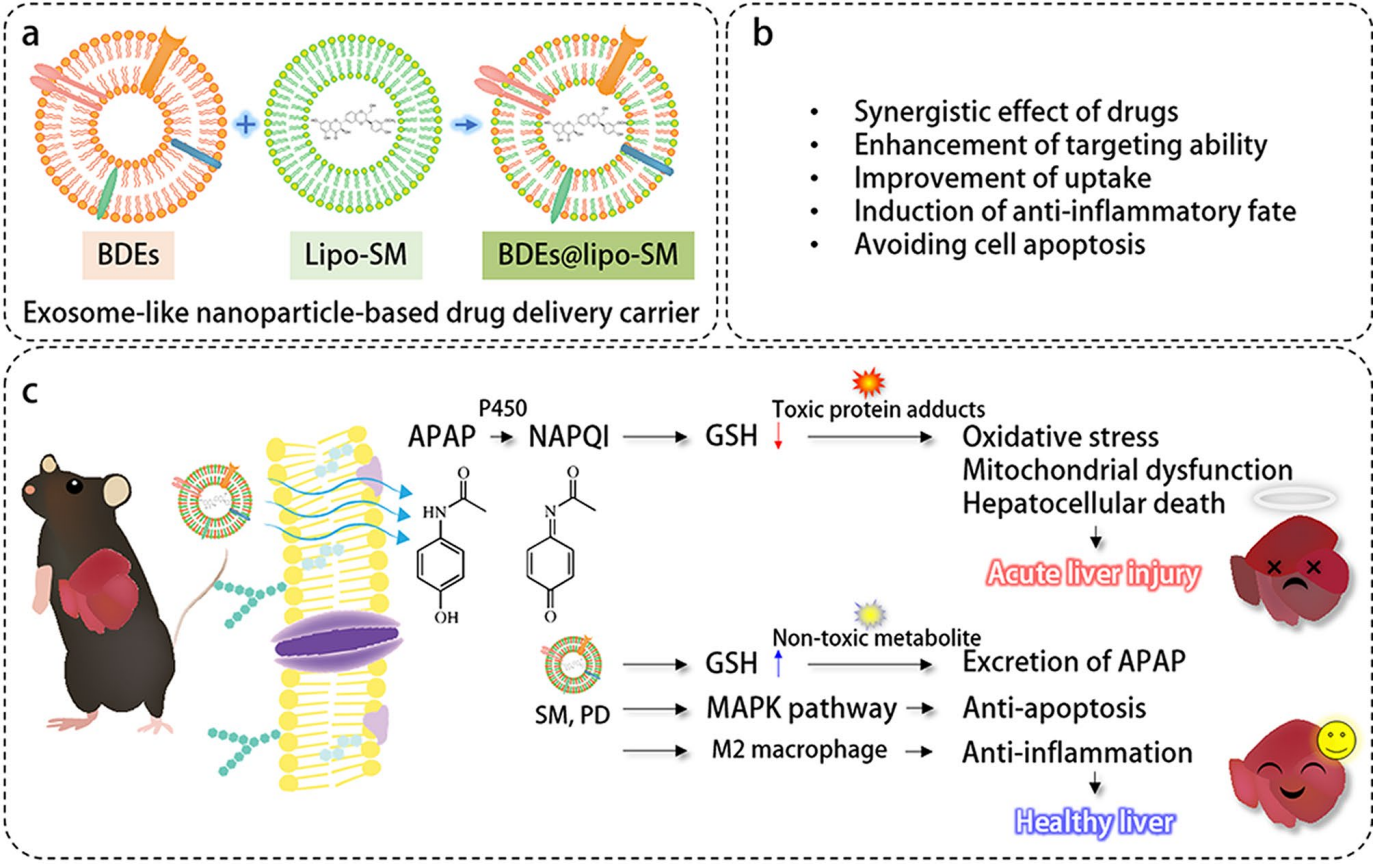
Molecular mechanism underlying ameliorative effects of BDEs@lipo-SM against APAP-induced cytotoxicity. (**a**) BDEs@lipo-SM was fabricated by thin film hydration and extrusion method. (**b**) Therapeutic benefits of BDE@lipo-SM in APAP-induced hepatotoxicity. (**c**) Acetaminophen (APAP) metabolic pathway and molecular mechanism of BDEs@lipo-SM in APAP-induced hepatotoxicity. APAP is bioactivated by cytochromes P450 to form a toxic intermediate, N-acetyl-p-benzoquinone imine (NAPQI). NAPQI is conjugated with gluthatione (GSH) resulting in non-toxic metabolites. During APAP overdose, a large amount of APAP is metabolized, which increases NAPQI generation depleting GSH. NAPQI-protein adducts in hepatocytes induce oxidative stress, mitochondrial dysfunction and cell death. BDEs@lipo-SM can increase GSH level and induce hepato-preventive and anti-apoptotic effect through MARK pathway

## Method and material

### Materials

Lecithin (Soya L-a-Phosphatidylcholine, SPC), Silibinin, acetaminophen (APAP), and Bortezomib were obtained from Meilun Biotechnology (Dalian, China) and used as received. Cholestrol was purchased from Sinopharm Chemical Reagent Co., Ltd. (Shanghai, China). 1,1’-dioctadecyl-3,3,3’,3’–tetramethylind-odicarbocyanine, 4-chlorobenzenesulfonate salt (DiD) was purchased from Fanbo Biochemical Co., Ltd. (Beijing, China). Dimethyl sulfoxide (DMSO) was purchased from Sigma-Aldrich (St. Louis, USA). Fetal bovine serum (FBS), Dulbecco’s modified Eagle’s medium (DMEM), phosphate buffer saline (PBS, pH 7.4), and trypsin were purchased from Yuanpei Biological Technology Co., Ltd (Shanghai, China). Mouse monoclonal antibodies were purchased from BioLegend (London, UK).

### Isolation of BDEs

100 g of *Platycodon grandiflorum*, harvested from Chifeng, Inner Mongolia, was purchased from a local market. For isolation of exosome-like nanoparticles, *Platycodon grandiflorum* was washed with a running water. Then, *Platycodon grandiflorum* was thoroughly ground in 300 mL 1X PBS with a blender for 5 min. The juice was squeezed on a sieve to remove rough debris and centrifuged at 2000 g for 20 min and the supernatant was further centrifuged at 10,000 g for 60 min. After centrifugation, the supernatant was collected and filtered using a 0.22 μm microporous filter membrane (Collins, USA). To isolate the BDEs, we used a tangential flow filtration method (TFF) using a Minimate EVO Tangential flow ultrafiltration system (Pall Biotech, Dreieich, Germany) to achieve high yield of the exosome-like nanoparticles without sucrose [16]. 200 mL of the multivesicular exosomal fluid was transferred into a feed tank and separated through the tangential filter, which is an Omega™ 500 kDa TFF Membrane (Pall Biotech, Dreieich, Germany), at the 20 mL/min of the feed flow rate and 2 bar of the transmembrane pressure. Particles larger than 100 nm concentrated were retained in the main vessel and particles smaller than 100 nm were in the filtrate vessel. While passing through the tangential filter, the final exosome-like nanoparticles were successfully diafiltered and concentrated about 6 times to reach a final volume of 30 mL of BDEs.

### Preparation of liposome loaded SM

The liposome loaded SM (lipo-SM) was prepared by thin-film hydration method. 25.5 mg SPC and 1.5 mg Cholesterol were dissolved in organic solvent (Chloroform : Methanol, 9:1, v/v). The solution was mixed with 1 mg SM dissolved in DMSO. Then, the organic solvent was removed using a rotary vacuum evaporation (ZX-98 rotary evaporator, LOOYE, China) for 10 min in a water bath at 48 ℃ to form a SM-containing lipid membrane. 500 µL of 1X PBS was added to the SM-containing lipid membrane and the lipid solution was stirred to get a preliminary nanoliposomal solution. The nanoliposomal solution was vortexed and sonicated using an ultrasonic cell pulverizer (20% amplitude, 2 s pulse on/off) for 2 min to turn the multilamellar lipid vesicles into smaller vesicles and the final nanoliposome was obtained by filtering through 0.22 μm filters.

### Fabrication of BDEs@lipo-SM

To promote hybrid BDEs with liposome loaded SM, the BDEs@lipo-SM was synthesized by simple thin film hydration followed by a membrane extrusion method. The fresh BDEs were added in the dry lipid layer to hydrate (1:1, v/v) in the final volume of 500 µL. Then, the mixture was then vortexed and sonicated at 20% amplitude for 2 s pulse on/off for 2 min. The formed multilamellar BDEs@lipo-SM was extruded through 800, 400, 200 and 100 nm size polycarbonate membranes, respectively, to achieve nano-sized unilamellar BDEs@lipo-SM. The final BDEs@lipo-SM was obtained by filtering through 0.22 μm filters [19, 20].

### Nanoparticle tracking analysis

Nanoparticle tracking analysis (NTA) was performed by NTA (NanoSight LM10, Malvern Instrument, UK). Size distribution and concentration of BDEs in liquid suspension were measured. BDEs were diluted to a final dilution of 1 : 10,000. 1 mL of BDEs was injected in the chamber with disposable sterile syringes (BD Discardit II, New Jersey, USA) until the solution came out at the end of the nozzle in a way. The sample was detected for 60 s duration using NanoSight automatic analysis settings at room temperature (RT). The average and SD values were analysed using the NTA 3.0 software (Malvern Instruments).

### Protein-based exosomes quantification of BDEs

To quantify BDEs, the protein concentration was measured by Bradford protein assay (Yeasen, China). The fresh BDEs were diluted for 10 times with 1X PBS and 5 mL of the diluted BDEs was used for measurement of the protein concentration. Bradford assay was performed as described in the previous report [21, 22].

### DLS measurement

The BDEs and fabricated BDEs@lipo-SM were diluted 100 times using diethylpyrocarbonate (DEPC) and 1 mL BDEs and fabricated BDEs@lipo-SM were transferred into a cuvette (12 mm square polystyrene cuvette, DTS0012, Malvern, UK) and a disposable cuvette (disposable capillary cell, DTS1070, Malvern, UK) for the size and zeta potential measurement, respectively. The cuvette was placed into the dynamic light scattering (DLS) device to start measuring and the size and surface charge were measured using a Zetasizer Nano ZS (Malvern Instrument, UK). The measurement was conducted at 25 ℃, and all experiments were run in triplicate.

### Encapsulation efficiency analysis by HPLC

The encapsulation efficiency of SM was determined by high-performance liquid chromatography (HPLC) (Agilent, USA). Drug loading capacity was calculated as SM encapsulated/liposome material×100%. To remove free SM in liposomes, the Lipo-SM was centrifuged at 64,000 g for 60 min at 4℃ with 10 mL 1X PBS. Then, the supernatant was removed and pellet was dissolved in 500 µL of an organic solvent consisting 10% Triton X and methanol (1:9, v/v). The sample was prepared by filtering through 0.22 μm filters for HPLC analysis. When performing HPLC analysis, SM was eluted through the column of Agilent ZORBAX SB-Aq 5 μm, 4.6 Х 250 mm (CA, USA) at a flow rate of 1 ml/min and separated using a mobile-phase gradient. (Mobile phase A; phosphate buffer (pH 5.0; 10 mM), mobile phase B; acetonitrile (ACN)) The analysis time was set for 22 min and the gradient was subsequently changed in mobile phase B to 29% at 0 min, 41% at 10 min, 29% at 20 min, and ended at 22 min. SM was analyzed using an Agilent 1260 Infinity II instrument (CA, USA). The drug encapsulation efficiency was calculated according to the following formula: The drug encapsulation efficiency = (total drug / free drug)/the initial added total drug × 100%.

### Cell culture

Huh7 liver cells and RAW 264.7 were purchased from Meilun Biotechnology (Dalian, China). All cells were cultured in DMEM with High glucose containing 10% FBS, 100 U/mL penicillin at 37 °C in a humidified atmosphere with 5% CO_2_. For subculturing, the culture media was removed and washed with PBS, followed by adding 0.25% Trypsin-EDTA. The cells were collected and centrifuged for 5 min at 1500 g. Cells were seeded on a 12 well plate at the density of 1 × 10^5^ for Huh7 and RAW 264.7 cell lines, respectively.

### Membrane fusion assay

To validate the fusion of membranes, a Fo¨rster resonance energy transfer (FRET) pair lipophilic dyes Coumarin 6 (C6, Ex/Em 460/510 nm) and Rhodamine B (RhB, Ex/Em 565/590 nm) were used. SPC and cholesterol in the mixture of chloroform and methanol (9:1, v: v) were incorporated with 8 µL 1 mg/mL of C6. The liposomal mixture was then evaporated to form a lipid film. The membranes of BDEs were labeled with 16 µL 1 mg/mL of Rhodamine B (RhB) by simple incubation for 10 min and subsequently washed twice to remove free dyes through UFC5100 Amicon Ultra-0.5 Centrifugal Filter Unit, 100 kDa (USA, Merck). C6 with lipid membranes (donor, acceptor) and RhB-labeled membrane of BDEs (acceptor) were applied at the ratio of 1:0, 1:0.25, 1:0.5, 1:0.75, 1:1, and 1:1.25, respectively followed by hydrating and extruding through 800, 400, 200, 100 nm polycarbonate filters to induce the membrane fusion. The fluorescence emission spectrum of each sample was obtained from 450 to 700 nm at an excitation wavelength of 430 nm on a Cary Eclipse fluorescence spectrophotometer (Agilent, USA).

### Preparation of DiD-labeled BDEs@lipo-SM

4 µL of 1 mg/mL of DiD-lipophilic dye, 250 µL of 1 mg/mL of BDEs and 50 µL of 1X PBS were mixed and incubated for 30 min at RT. Consistently, the mixture was centrifuged using UFC5100 Amicon Ultra-0.5 Centrifugal Filter Unit, 100 kDa to remove free DiD-lipophilic dye. Fluorescence intensity of DiD-labeled BDEs was measured at 2–3 × 10^10^ using In Vivo Imaging System Spectrum (IVIS). When preparing DiD-labeled BDEs@lipo-SM, 4 µL of 1 mg/mL of DiD-lipophilic dye was additionally added during preparation of liposome loaded SM. Then, after evaporation of the organic solvent, the dry lipid film is rehydrated with 500 µL of 1X PBS and finally the DiD-labeled lipid solution was achieved. Consequently, DiD-labeled BDEs@lipo-SM was prepared by a membrane extrusion method as mentioned above.

### In vitro drug release kinetics of SM from BDEs, liposomes, and BDEs@lipo

In vitro drug release study was performed to determine the percentage of SM released from BDEs, liposomes, and BDEs-lipo. The in vitro drug release study was carried out using dialysis membrane (MW 12,000–14,000 Da), that allows the diffusion of free SM. The samples were were placed in the dialysis membrane bags and the dialysis membrane bags were put in glass vials with 100 mL of 1X PBS. The samples were gently rocked at 50 rpm and 37 ℃ during the process. Cumulative drug-release kinetics of SM from BDEs, liposomes, and BDEs-lipo were measured at the different time points for 24 h. At predetermined time points of 0, 1, 2, 4, 8, 12, 16, 20, and 24 h, the samples were collected and cumulative SM release was analyzed using a UV spectrophotometer at λ_max_ 286 nm. The samples were replaced in the glass vials with the same volume of fresh buffer. The percentage of SM released was determined as follows:

$$Release \left(\%\right) = \left\{\right[SM] released/[SM\left] total\right\} \times 100$$

### Analysis of cellular uptake of BDEs@lipo-SM by flow cytometry

To determine cellular uptake of BDEs@lipo-SM, Huh7 and RAW 264.7 cells were seeded on a 12 well plate at the density of 1 × 10^5^. The cells were incubated for 24 h and subsequently 20 µL of DiD-labeled BDEs@lipo-SM was conducted in the cells. The cells were incubated at 37℃ for 1, 3, and 6 h. Then, the cells were collected for detection of the internalized fluorescence of BDEs@lipo-SM by flow cytometry.

### Immunostaining and flow cytometry

To analyze the M2 macrophage proliferation by the macrophage markers in RAW 264.7 cells, flow cytometry was performed. RAW 264.7 cell line was seeded in a 12 well plate at 1 × 10^5^ cells/well. After incubation for 24 h, BDEs and BDEs@lipo-SM were added in the cells and the plate was incubated for 48 h. Non-modified BDEs or BDEs@lipo-SM were considered as a control. Then, the media was removed and cells were collected after washing with 1X PBS. The cells were blocked with 3% BSA on ice for 30 min and centrifuged at 1500 g for 5 min at 4 ℃. Then, the supernatant was removed and the cells were resuspended with 1X PBS and incubated with an eFluore 450-labeled anti-CD45 mAb, PE-labeled anti-CD86 mAb, and APC-labeled anti-CD206 mAb (dilution, 1:100) for 30 min at RT avoiding light. Subsequently, the cells were washed 3 times with 1X PBS and detected on The Cytomics™ FC 500 Flow Cytometry (Beckman Coulter, Brea, CA, USA). The result was organized by FlowJo software (BD biosciences, USA).

### Flow cytometry assay using annexin V/PI staining

To evaluate cell apoptosis of BDEs@lipo-SM, apoptosis was detected using an FITC-Annexin V apoptosis detection kit (BD Pharmingen, USA). RAW 264.7 cells were seeded at the density of 1 × 10^5^ cell/well in a 12-well plate and incubated for 24 h before treatment. After 48 h of treatment of BDEs@lipo-SM at concentrations of from 0 to 200 µg/ml, both treated and untreated cells were trypsinized and collected. Then, the cells were suspended in 1X binding buffer and incubated with 2.5 µL of fluorescein isothiocyanate (FITC)-conjugated Annexin V and 5 µl of phycoerythrin (PE)-conjugated PI for 15 min in the dark at RT followed by flow cytometry.

### RT-PCR

Total RNA was isolated from Huh7 and RAW 264.7 cells treated with a serial dilution of BDEs@lipo-SM ranging from 1 µg/mL to 50 µg/mL for 48 h. cDNA was prepared from the total RNA and consistently RT-PCR was performed using Hifair III One Step RTqPCR SYBR Green Kit (Yeasen, China) according to the manufacturer’s instructions. Primer sets are described in Table 1. To determine apoptosis of immune cells, apoptosis and inflammatory-related genes were examined including BAX, IL-6, IL-1b, TNF-α and the reference gene, GAPDH. To evaluate the gene expression of the targets, the solution containing DNA polymerase, primer pair and cDNA was pre-heated for 5 min at 95˚C to activate and denature non-specific DNA binding. Immediately, the reaction was performed by 40 cycles of 95˚C for 20 s, 55˚C for 30 s and 68˚C for 20 s using QuantStudio 3. Relative gene expression was calculated as the ΔΔCT method and normalized to the expression of GAPDH as a standard for gene expression quantification. All qRT-PCRs were performed in triplicate, and the data are presented as means ± standard errors of the means (S.E.M).

### Western blot analysis

The cells were washed with 1X PBS on ice. Then, the ice-cold RIPA buffer including protease and phosphatase inhibitor was added in the cells and the cells were scraped off and the cell suspension was transferred into a 1.5mL microcentrifuge tube. The tube was centrifuged at 14,000 g at 4 °C for 10 min. The supernatant was transferred to a fresh tube. Cell lysates were analyzed for total protein concentration using the BCA protein assay kit (Beyotime, Shanghai, China). 25 µg of total protein were loaded on 10% acrylamide gel. Relative enzyme levels were quantified using antibodies; mouse mAb p-ERK 1/2; ERK 1/2; p-JNK; JNK; p-p38; p38.

### Measurement of AST/ALT level

To evaluate the liver damage, after treatment of BDEs@lipo-SM, the blood was collected and placed at room temperature for 2 h to separate the serum sample. The blood was then centrifuged at 1000 g for 15 min and the supernatant was taken followed by stored at -80 ℃. Serum aspartate aminotransferase (AST) and alanine aminotransferase (ALT) levels were assessed in blood plasma by Wuhan Servicebio Technology Co., Ltd. (Wuhan, China) using Chemray 240 automatic biochemical analyzer (Rayto, Shenzhen, China).

### Animal studies and ethical approval of animal experiment

18–20 g of male C57BL/6J mice at the age of 6–7 weeks were purchased from Sino-British SIPPR/BK Lab. Animal Co., Ltd. (Shanghai, China) and housed under pathogen-free conditions. The animal experiments were conducted at School of Pharmacy in Fudan University in accordance with the Guiding Principles for the Care and Use of Experimental Animals (Shanghai, China). To induce hepatotoxicity, the mice were injected intraperitoneally with 500 mg/kg of APAP and right after treated with 200 µL of 1 mg/mL of BDEs, 150 mg/kg NAC, and 200 µL of 1 mg/mL of BDEs@lipo-SM (based on the BDEs concentration) once. Before being treated, mice were fasted for 24 h. After 24 h of administration, the mice were anesthetized and humanly sacrificed by cervical dislocation and the liver was collected for subsequent analysis.

### Statistical analysis

Statistical significance was determined using the t-test, and a p-value < 0.05 was considered statistically significant, and non-significant result was recorded as N.S. The t-test was conducted through GraphPad Prism 7.0 software (CA, USA).

## Results

### Isolation and characterization of BDEs

**Fig. 1:**
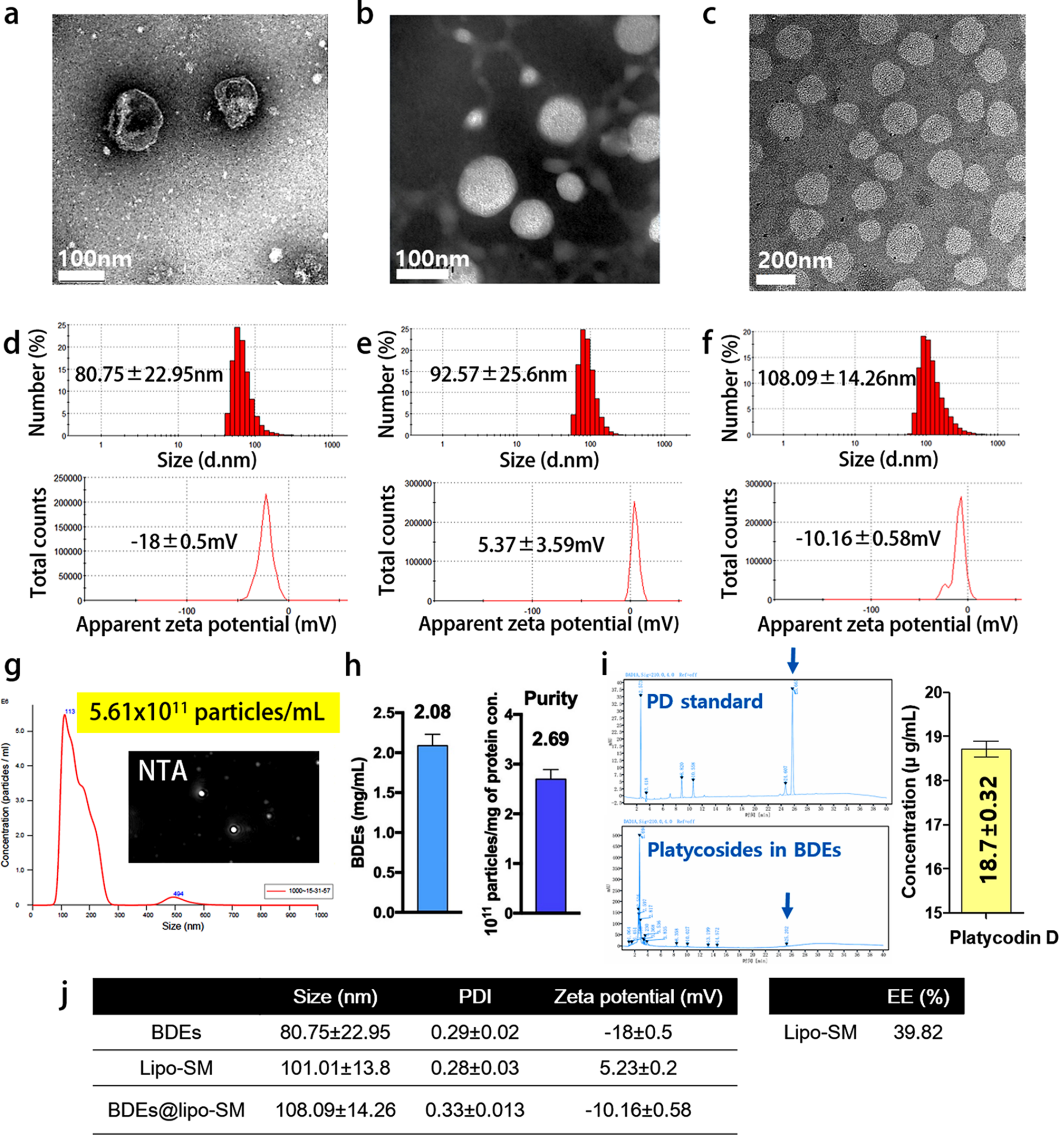
Characterization of BDEs and BDEs@lipo-SM. (**a**-**c**) The round-shaped morphology of BDEs, liposomes, and BDEs@lipo was observed by transmission electron microscopy (TEM). (**d**-**f**) Size distribution and zeta potential were determined with a serial dilution of BDEs, liposomes, and BDEs@lipo by dynamic light scattering (DLS). (**g**) Platycodin D in BDEs was evaluated by HPLC analysis. (**h**) Tabular form of the size, PDI, and zeta potential of BDEs, liposomes-loaded SM, and BDEs@lipo-SM, respectively. (**i**) An encapsulation efficiency (EE) of SM in liposomes was determined

To isolate BDEs with high purity grade and yield, BDEs were obtained using the TFF method. After differential centrifugation of a homogeneous balloon flower root juice, the multivesicular exosomal fluid was collected and exposed to a Tangential flow ultrafiltration system. The fluid was separated through the tangential filter and the filtrate containing particles < 100 nm was collected in a separate vessel. The final filtrate of exosome-like nanoparticles had a 6-fold higher concentration of BDEs. The morphology of BDEs was observed by TEM, as shown in Fig. 1a, confirming the isolation of 100 nm spherical nanovesicles enclosed by a bilayer lipid membrane without aggregation or degradation. In addition, the morphology of liposomes and BDEs@lipo were observed by TEM. TEM images provided evidence for the homogenous spherical shape with an average particle size about 100 nm and 200 nm for the liposomes and BDEs@lipo, repectively (Fig. 1b, c). DLS measurements based on number were performed to determine the most abundant size population. Similar to the TEM results, the average diameter of BDEs, liposomes, and BDEs@lipo was approximately 80.75 ± 22.95, 92.57 ± 25.6, and 106.8 ± 14.24 nm with a low polydispersity index (PDI). The surface charge of the BDEs indicated that the BDEs were covered by a negative charge of − 18 ± 0.5 mV. After the fusion of the membrane, the surface charge of BDEs@lipo became less negatively charged of -10.8 ± 6.46 mV. This decrease of the surface charge could indeed be attributed to the positive charge contribution from the liposomes (Fig. 1d-f).

To clearly define the content of the bioactive component of BDEs, we performed an HPLC analysis. After extraction, the evaluation of the overall chemical compositions of BDE revealed an enrichment of platycodin D, which is believed to contribute to the efficacy of BDEs in inflammation and related conditions (Fig. 1g).

### Preparation and characterization of BDEs@lipo-SM

To develop an effective therapy for APAP-induced hepatotoxicity, we designed hybrid BDEs with SPC liposome-loaded SM (BDEs@lipo-SM). To fabricate BDEs@lipo-SM, SPC-based liposomes were prepared for the encapsulation of SM using a thin-film hydration method. After fabrication of the SPC-based liposomes loaded with SM, BDEs were hybridized with the liposomes by a membrane extrusion method. The BDEs@lipo-SM were then examined using DLS. The average particle size of BDEs@lipo-SM obtained from the DLS was 108.09 ± 14.26 nm. The zeta potential was also determined to be -10.16 ± 0.58 mV. After hybridization, the average particle size increased slightly compared to BDE, and it appeared that the SPC-based liposome was larger than the BDE. Similar to the change in size distribution, the negative zeta potential of BDEs@lipo-SM was slightly increased. It was assumed that this increase in size also contributed to the increase in surface charge of the SPC-based liposome (Fig. 1h).

#### Evaluation of encapsulation efficiency

To demonstrate the efficacy of the SPC-based liposomes loaded with SM, we first optimized the formulation conditions [20]. Under our experimental conditions, the optimized formulation with SPC and cholesterol content showed the best encapsulation and integrity with good storage stability (Data not shown). Then, the encapsulation efficiency of lipo-SM was further determined by HPLC, using the ratio of encapsulated SM to the initial added SM. The encapsulation efficiency was estimated to be approximately 39.82% (Fig. 1i).

#### Comparison of total yield of BDEs isolated by UC and the TFF method

When isolating BDEs, unlike the previous studies of plant-derived exosomes-like nanoparticles, BDEs were isolated by the TFF method. The comparison between BDEs isolated by UC and the TFF method revealed differences in the average size distribution. BDEs isolated by UC had an average size of approximately 85.02 ± 31.93 nm, while those isolated by TFF were slightly smaller, with an average size of about 74.46 ± 31.25 nm. This indicates that the choice of isolation method can influence the size distribution of the isolated nanoparticles, with TFF resulting in slightly smaller BDEs compared to UC (Figure. S1a, b). However, practically, the size distribution of them was not significantly different.

The evaluation of size distribution and particle number using NTA revealed that the concentration of particles obtained was 5.06 × 10^11^ particles/mL for UC and 5.61 × 10^11^ particles/mL for the TFF method. This suggests that the TFF method is more efficient in isolating BDEs because it resulted in a higher number of particles per unit volume compared to UC. This higher particle concentration indicates that TFF might be a more effective method for isolating BDEs in terms of particle yield (Figure. S1c, d).

Furthermore, the yield of BDE isolated by two different methods was quantified by the total protein concentration through Bradford protein quantification analysis. The protein concentration of BDEs was approximately 3.65 ± 0.4 mg/mL for UC and 2.08 ± 0.2 mg/mL for TFF. The purity of BDEs was calculated by determining the ratio of particle counts to protein concentration. BDEs isolated by UC had a purity of approximately 1.38 ± 63 × 10^11^ particles/mg of protein concentration, while those isolated by the TFF method exhibited a higher purity of about 2.69 ± 0.11 × 10^11^ particles/mg of protein concentration (Figure. S1e, f).

Based on these purity values, the relative total yield of BDEs isolated by TFF was deduced by multiplying the final volume of BDEs by the protein concentration. The results showed that the increased relative total yield for TFF was 2.8 times higher than that for UC. This indicates that not only does TFF yield a higher number of particles per unit volume, but it also results in BDEs with a higher purity, leading to a significantly increased relative total yield compared to UC (Figure. S1g).

Overall, while both methods yielded similar size distributions of BDEs, the UC method led to increased protein concentration due to particle aggregation during sequential UC processes. This aggregation might result in lower purity and total yield compared to the TFF method. On the other hand, the TFF method appears to offer advantages in terms of both purity and yield of BDEs. Since there’s no mention of particle aggregation in TFF, it likely produces BDEs with less protein contamination, leading to higher purity. Additionally, it seems the TFF method yields a greater quantity of BDEs compared to UC, likely due to its efficiency in isolating and concentrating the particles without significant aggregation issues.

In summary, while both methods may produce BDEs with similar size distributions, the TFF method seems to offer superior purity and yield, making it a more favorable choice for isolating BDEs.

#### Membrane fusion of BDEs and liposomes

To evaluate membrane fusion between BDEs and liposomes, Förster Resonance Energy Transfer (FRET) and microscopy imaging techniques were employed. In the FRET process, the lipid membranes of the BDEs were labeled with RhB, which acts as the acceptor fluorescent molecule. Conversely, the liposomes were loaded with C6, serving as the donor fluorescent molecule. For effective FRET to occur, the donor (C6) and acceptor (RhB) fluorescent dyes must be in close proximity, typically within approximately 10 nm. This close spatial arrangement allows the energy transfer from the excited donor to the acceptor. Specifically, the absorption spectrum of RhB overlaps with the emission spectrum of C6. As a result, when C6 is excited, it transfers energy to RhB, leading to a decrease in the fluorescence intensity of C6 and a corresponding increase in the fluorescence intensity of RhB (Figure. S2a, b). As shown in Figure. S2b, the fluorescence intensity of BDEs@lipo exhibited an increase in the fluorescence signal at 590 nm (corresponding to RhB) and a decrease at 495 nm (corresponding to C6) with increasing input of BDEs. This observation confirms that membrane fusion between BDEs and liposomes brings the donor (C6) and acceptor (RhB) molecules into close proximity, facilitating efficient energy transfer. The quantifiable changes in fluorescence intensities at these specific wavelengths are indicative of the extent of fusion, with the increased fluorescence of RhB at 590 nm and the decreased fluorescence of C6 at 495 nm serving as key markers of this process.

Microscopy images provide visual confirmation and further insights into the fusion process, complementing the FRET data. As shown in Figure. S2c, the colocalization of BDEs@lipo was observed in RAW 264.7 cells. Before extrusion, DiD-labeled BDEs (green) and DiR-labeled liposomes (red) were clearly separated and unincorporated, as indicated by their distinct, non-overlapping fluorescence signals. After extrusion, however, DiD-labeled BDEs and DiR-labeled liposomes showed significant overlap of their fluorescence signals, indicating that the two components had successfully fused. This colocalization was observed as a merging of the green and red signals, confirming that the BDEs and liposomes had incorporated into a single entity. These microscopy findings corroborate the FRET data, where the increase in RhB fluorescence and decrease in C6 fluorescence also indicated successful membrane fusion. Together, these methods provide robust evidence of the effective fusion of BDEs and liposomes.

### Stability and in vitro drug release kinetics of SM

To evaluate the stability of BDEs@lipo, the size distribution was measured over 7 days using DLS. BDEs, liposomes, and BDEs@lipo were prepared and placed in 1X PBS at room temperature. The size distribution of BDEs, liposomes, and BDEs@lipo remained in the range of approximately 50 to 120 nm throughout the observation period (Figure. S3a). Although the average size of the nanovesicles showed a slight increase over time in all samples, the change was not significant. This indicates that the nanovesicles maintained their stability in terms of size distribution over the 7-day period, suggesting that BDEs@lipo are stable under the tested conditions.

As shown in Figure S3b, the dissolution profiles of SM were evaluated in a phosphate buffer with a pH of 7.4, revealing distinct release patterns for different formulations over 24 h. Initially, the drug release from SM formulated in BDEs, liposomes, and BDEs@lipo followed similar trends for the first 8 h. However, beyond this period, the SM formulated in BDEs@lipo demonstrated a sustained release, reaching 34.08 ± 9.2% at the 24 h mark. While the SM encapsulated in BDEs and liposomes showed comparably faster release rates, achieving 39.42 ± 3.46% and 41.06 ± 14.89% respectively by the end of the 24 h period.

This slow and sustained release profile of SM from the BDEs@lipo formulation suggests enhanced drug encapsulation and controlled release, likely due to physical and chemical interactions between the drug, phospholipids, and liposomes. These interactions increase the drug’s encapsulation efficiency compared to its free form, as indicated by in vitro solubility studies. Consequently, the BDEs@lipo formulation appears to offer a more efficient and sustained release mechanism for SM, making it a promising option for achieving prolonged drug release in physiological conditions.

### Cellular uptake of BDEs and BDEs@lipo-SM in vitro

**Fig. 2:**
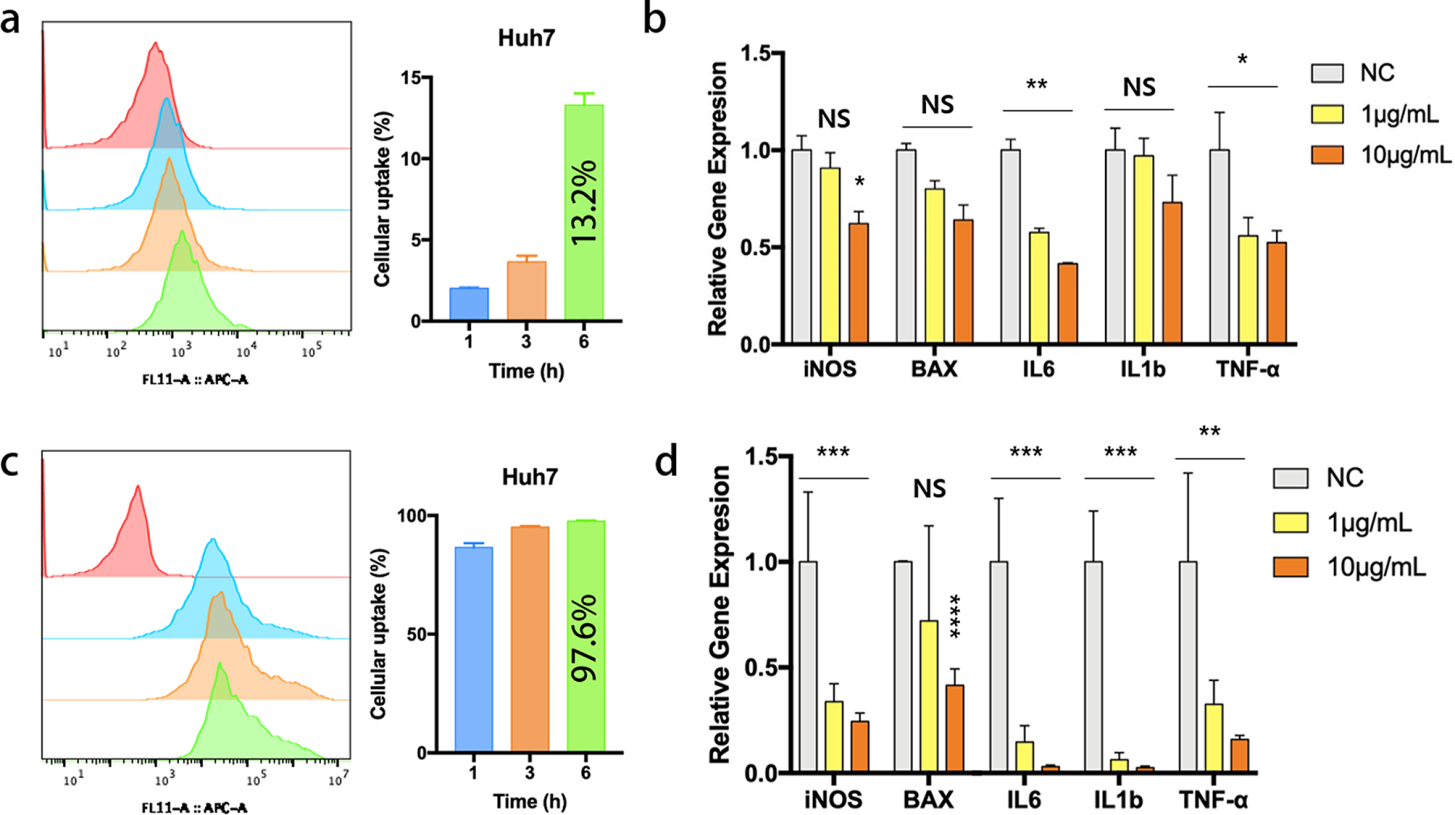
Cellular uptake of BDEs and BDEs@lipo-SM in hepatocyte and gene expression of apoptosis and inflammatory related genes. (**a**) The representative histograms and cellular uptake of DiD-labeled BDEs in Huh7 cells was determined at the different time points using flow cytometry analysis. (**b**) Gene expression of pro-apoptotic related genes including iNOS, BAX, IL6, IL1b, and TNF-α in Huh7 cells in the presence of 1 and 10 µg/mL BDEs. (**c**) The representative histograms and cellular uptake of DiD-labeled BDEs@lipo-SM in Huh7 cells was determined at the different time points using flow cytometry analysis. (**d**) Gene expression of pro-apoptotic related genes including iNOS, BAX, IL6, IL1b, and TNF-α in Huh7 cells in the presence of 1 and 10 µg/mL BDEs@lipo-SM. (*P < 0.05, **P < 0.01, ***P < 0.001, ****P < 0.0001) All the relative gene expressions are normalized to GAPDH mRNA expression

A key factor in the development of drug delivery technologies with lipid-based systems is their capacity for internalization by target cells. Thus, DiD-labeled BDEs and BDEs@lipo-SM were evaluated for cellular uptake via flow cytometry. The cellular uptake of DiD-labeled BDEs increased for up to 6 h in Huh7 cells, achieving 13.2%±2.12, nonetheless, it was not considered very efficient (Fig. 2a). In Huh7 cells exposed to DiD-labeled BDEs@lipo-SM, the cellular uptake of DiD-labeled BDEs@lipo-SM increased to 97.6%±0.75 at 6 h. This increase indicates that the internalization efficiency of the preparation was approximately 7.3-fold higher that achieved by cells in the presence of DiD-labeled BDEs (Fig. 2c).

## Regulation of apoptosis and inflammation

To evaluate whether BDEs and BDEs@lipo-SM can regulate apoptosis and inhibit the inflammatory response, the level of expression genes related to apoptosis and to inflammatory inflammation was analyzed by RT-qPCR. Huh7 cells were exposed to 1 and 10 µg/mL BDEs and BDE@lipo-SM treatment for 48 h and the apoptosis-related gene, BAX, and the inflammatory markers iNOS, IL6, IL1b, and TNF-α were quantified. As shown in Fig. 2b and d, BAX expression (a pro-apoptotic factor) decreased significantly following treatment with both BDE and BDE@lipo-SM. Thus, BDEs and BDEs@lipo-SM can induce an antiapoptotic effect on Huh7 cells. A similar trend of down-regulation of inflammatory-related genes was observed following treatment with BDE and BDEs@lipo-SM. However, treatment with BDEs@lipo-SM exhibited a stronger anti-inflammatory effect than treatment with BDEs, as evidenced by the greatly reduced level of gene expression of inflammatory markers (Fig. 2b and d). Because well-fabricated BDEs@lipo-SM intrinsically contain natural bioactive molecules, such as PD that can successfully encapsulate SM and be delivered in cells, inducing significant anti-apoptosis and inflammatory effects. Thus, BDEs@lipo-SM could be utilized as drug delivery agents to enhance therapeutic efficacy.

### Proliferation of M2 macrophages

**Fig. 3:**
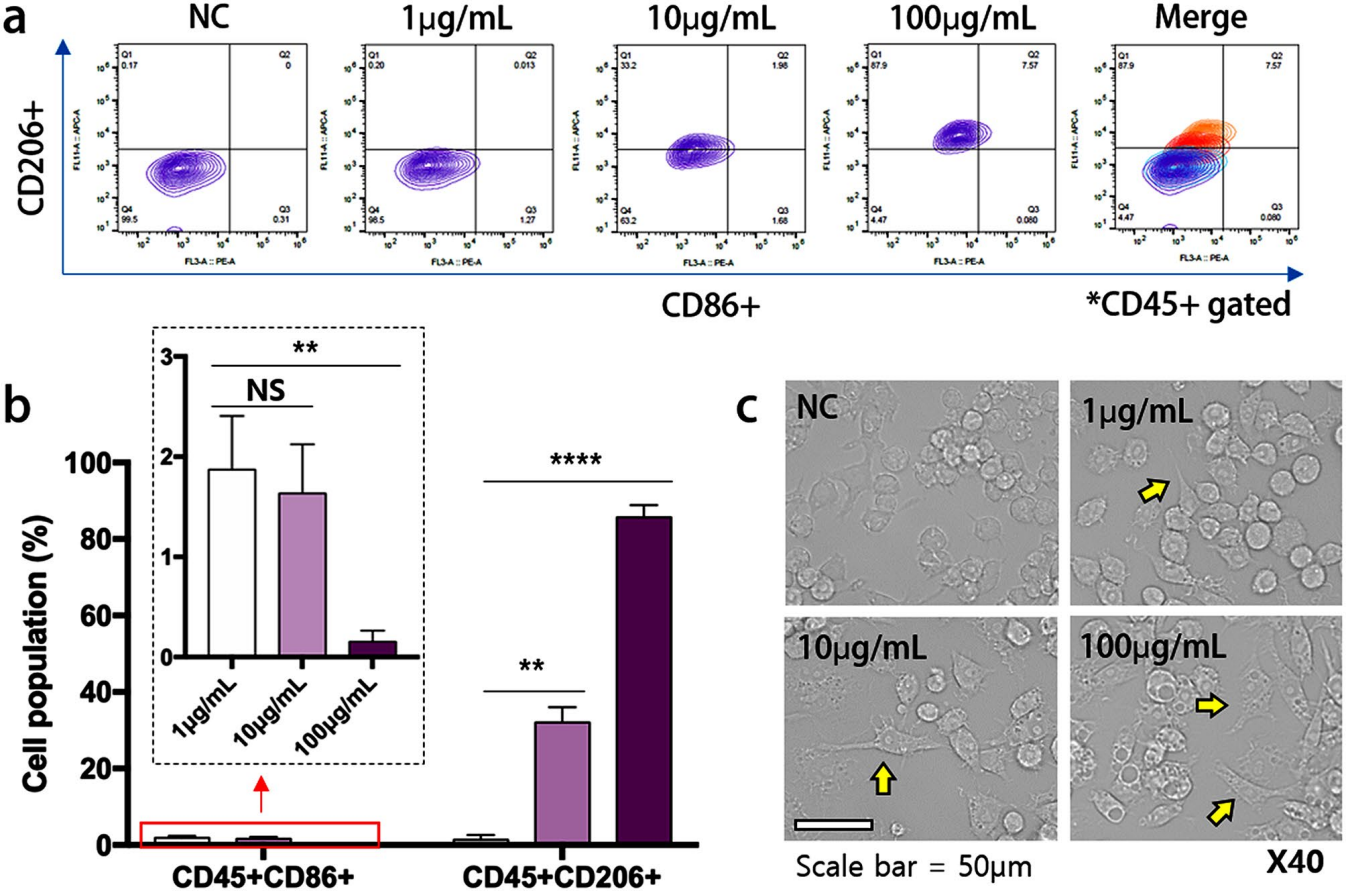
Promotion of M2 macrophage of BDEs@lipo-SM in RAW 264.7 cells. (**a**) RAW 264.7 cells were proliferated into M2 macrophages with treatment of BDEs@lipo-SM. (**b**) Evaluation of cell population of M1 (CD45+, CD86+) and M2 (CD45+, CD206+) macrophages. (**c**) The morphological differences of macrophages in M2 macrophage with treatment of BDEs@lipo-SM. M2 polarized macrophages had an elongated and stellate morphology

To evaluate whether BDEs@lipo-SM can induce anti-inflammatory reactions in the macrophage, we treated RAW 264.7 cells with BDEs@lipo-SM, and the effects of BDEs@lipo-SM on macrophage polarization cells was analyzed by flow cytometry. As shown in Fig. 3, the M2 macrophage marker, CD206, markedly increased in the presence of BDEs@lipo-SM, while the M1 macrophage marker, CD86, and levels of pro-inflammatory cytokines, did not increase and even decreased in a concentration-dependent manner (Fig. 3a and b). Interestingly, the M2 macrophage marker CD206 was markedly up-regulated after treatment with 100 µg/mL BDEs@lipo-SM to 91 ± 1.52%, while the M1 macrophage marker CD86 decreased to 0.23 ± 0.17%. Furthermore, macrophage morphological changes were observed in M2 macrophages at different concentrations of BDE@lipo-SM under an inverted phase contrast microscope. The cells treated with free BDEs@lipo-SM showed mainly homogeneous rounded-shaped morphology, whereas cells treated with BDEs@lipo-SM appeared as a heterogeneous cell population with variable pleomorphic morphology. The elongated cell bodies with cytoplasmic extensions were slightly larger than those of the untreated cells **(**Fig. 3c).

### In vivo biodistribution of BDEs@lipo-SM

**Fig. 4:**
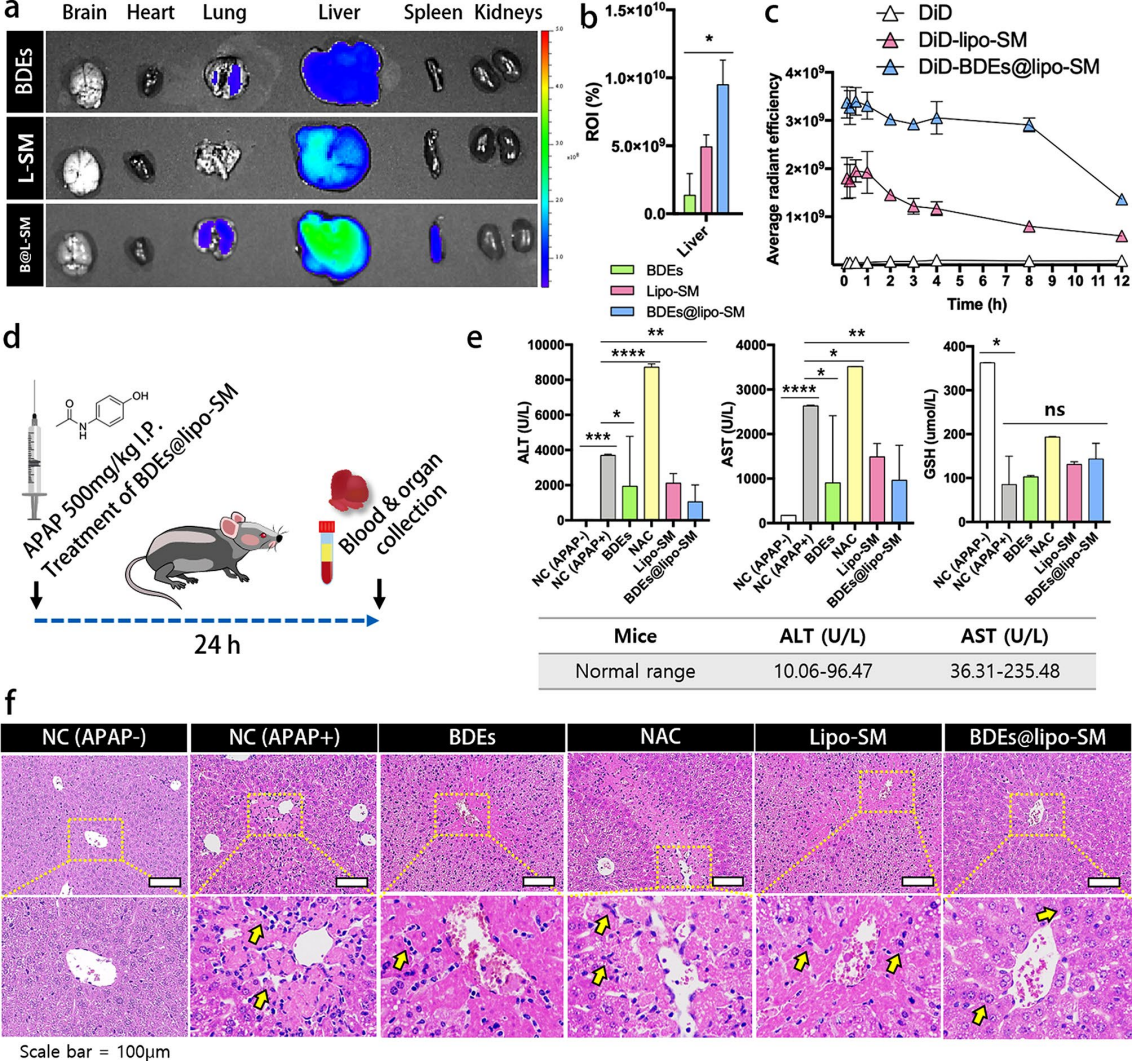
Alleviate effect of BDEs@lipo-SM in APAP-induced liver toxicity at doses of 500 mg/kg in vivo assay and pharmacokinetics study of BDEs@lipo-SM. (**a**) Biodistribution of DiD-labeled BDEs, DiD-labeled lipo-SM, and DiD-labeled BDEs@lipo-SM. In vivo imaging system (IVIS) was used to image brain, heart, liver, spleen and kidneys. (**b**) Increased liver accumulation of DiD-labeled BDEs@lipo-SM by approximately 2 folds compared to lipo-SM. (**c**) Average radiant efficiency of blood in mice after treatment of DiD-PBS, DiD-labeled lipo-SM, DiD-labeled BDEs@lipo-SM at the indicated time points (0.125, 0.25, 0.5, 1, 2, 3, 4, 8 and 12 h) post injection. (**d**) Schematic illustration of APAP-induced hepatotoxicity in vivo assay. (**e**) Blood chemistry test of AST, ALT and GSH to indicate liver toxicity. (**f**) H&E staining of liver tissue obtained from mice with non-treated APAP, APAP, APAP + BDEs, APAP + NAC, APAP + lipo-SM and APAP + BDEs@lipo-SM. (Upper panel) High magnification view of area presented in yellow box of (f) (Lower panel)

To validate the biodistribution of BDEs@lipo-SM in vivo, the main organs including the brain, heart, liver, spleen, and kidneys were collected from C57BL/6j mice and analyzed by the IVIS imaging system after 24 h of intravenous administration of DiD-BDEs, DiD-lipo-SM, and DiD-BDEs@lipo-SM. As shown in Fig. 4a, DiD-BDEs@lipo-SM were mainly found in the liver and redistributed to the lung and spleen. The detailed quantitative fluorescence values of the IVIS images showed that the accumulation of DiD-BDEs@lipo-SM in the liver was significantly increased by approximately 2-fold compared to DiD-lipo-SM, showing a more prominent hepatic accumulation than DiD-BDEs and DiD-lipo-SM (Fig. 4b). DiD-BDEs@lipo-SM displayed effective delivery to the liver, indicating that BDEs@lipo-SM could be an attractive vehicle for a drug delivery system targeting drugs to the liver.

### In vivo imaging of the stability and long-term accumulation of BDEs@lipo

The in vivo stability and long-term accumulation of BDEs@lipo were evaluated by measuring fluorescent intensity over time in vivo. For this purpose, DiD-labeled BDEs, liposomes, and BDEs@lipo were intravenously injected into 6-7-week-old nude mice. The fluorescence intensity was measured using an IVIS at various time points over a 24 h period (as depicted in Figure S4).

The results demonstrated that BDEs@lipo exhibited significantly higher fluorescence intensity across all time points compared to BDEs and liposomes alone. This indicates that BDEs@lipo accumulated rapidly and maintained higher levels of fluorescence throughout the entire observation period. These findings suggest that BDEs@lipo are stable in vivo and possess a prolonged circulation time, thereby reducing rapid elimination from the body. This enhanced stability and extended circulation time imply that BDEs@lipo could offer considerable advantages for in vivo applications, potentially leading to improved therapeutic outcomes.

### Blood pharmacokinetics

After confirming the biodistribution of BDEs@lipo-SM, we further performed serum pharmacokinetic studies to evaluate the retention time of SPC-based liposomes and BDEs@lipo-SM in vivo in the blood circulation. Blood samples were collected at different time intervals (0.125, 0.25, 0.5, 1, 2, 3, 4, 8, and 12 h) after a single intravenous injection of DiD-PBS, DiD-lipo-SM, and DiD-BDEs@lipo-SM, respectively; DiD-PBS was used as a control. The average fluorescence emission was determined and quantified using the IVIS imaging system. For the hematic pharmacokinetic studies, we first confirmed the same fluorescence intensity of DiD-lipo-SM and DiD-BDEs@lipo-SM to ensure equal analysis conditions (Data not shown). As shown in Fig. 4c, DiD-BDEs@lipo-SM achieved a prolonged circulation time in the blood. Furthermore, an evident bright fluorescence signal for DiD-BDEs@lipo-SM could still be observed in the blood sample 12 h after injection, indicating that hybrid BDEs could effectively escape drug clearance by the reticuloendothelial system (RES) and thus obtain enhanced blood retention in vivo compared to liposomes. Pharmacokinetic studies showed BDEs@lipo-SM exhibited significantly longer half-life and slower clearance compared to liposomes. In summary, BDEs@lipo-SM would enable enhanced targetability in the liver with no toxicity and possess a prolonged half-life. These results suggest that treatment with BDEs@lipo-SM is safe for APAP-induced hepatotoxicity and provides opportunities for recovery from liver damage after a single treatment.

### Effects on alleviation of APAP-induced hepatotoxicity in vivo

To evaluate the improvement of APAP-induced hepatotoxicity, blood biochemistry tests were performed to measure serum alanine aminotransferase (ALT), aspartate aminotransferase (AST), and GSH levels. After intraperitoneal injection of 500 mg/mL of APAP, BDEs, NAC, lipo-SM, and BDEs@lipo-SM were injected intravenously and serum and liver markers were evaluated for each mouse after 24 h of injection (Fig. 4d). As shown in Fig. 4e, after BDE treatment @lipo-SM, ALT, and AST levels decreased significantly compared to the APAP-treated group. Treatment with NAC, a glutathione precursor, an antidote for APAP overdose, produced significantly decreased serum conditions of ALT and AST 24 h after treatment.

In the course of APAP hepatotoxicity, GSH depletion plays a pivotal role in inducing oxidative stress [23–25]. Thus, it is important to restore GSH levels to overcome long-term damage to liver tissue. GSH levels showed a similar trend as that observed for ALT and AST levels. GSH levels were significantly increased in the treatment of BDEs@lipo-SM compared to the APAP-treated group. Overall, these results suggest that BDEs@lipo-SM allowed to restore GSH levels, which can counteract oxidative stress conditions; thus, attenuating liver injury and inducing liver regeneration [26–28].

### Histopathological findings of APAP-induced hepatotoxicity

Consistently, based on the results of the blood chemistry evaluations, we further performed the histopathological evaluation of liver tissues from BDEs@lipo-SM-treated mice after 24 h and compared tissues of control mice without exposure to APAP, and exposed to APAP, BDEs, NAC, and lipo-SM to verify the degree of severity of APAP-induced hepatotoxicity. As shown in Fig. 4f, after a 24 h treatment, liver tissue in BDEs@lipo-SM treated mice showed comparable intact liver structure with round liver cells and less inflammatory infiltration or cell necrosis surrounding the central veins of the liver, whereas hepatocyte disruption was observed in areas of severe sinusoidal congestion accompanied by extensive centrilobular necrosis, loss of hepatocyte nuclei, and hemorrhaging in the APAP treated group and in the other experimental groups. (Yellow arrow) The results of these pathological changes indicated that treatment with BDEs@lipo-SM can alleviate inflammation and protect the liver, as revealed by decreases in hemorrhage and inflammation infiltration and recovery in hepatocytes.

### Mechanism of antihepatotoxicity of BDEs@lipo-SM in the MAPK/ERK 1/2 pathway

**Fig. 5:**
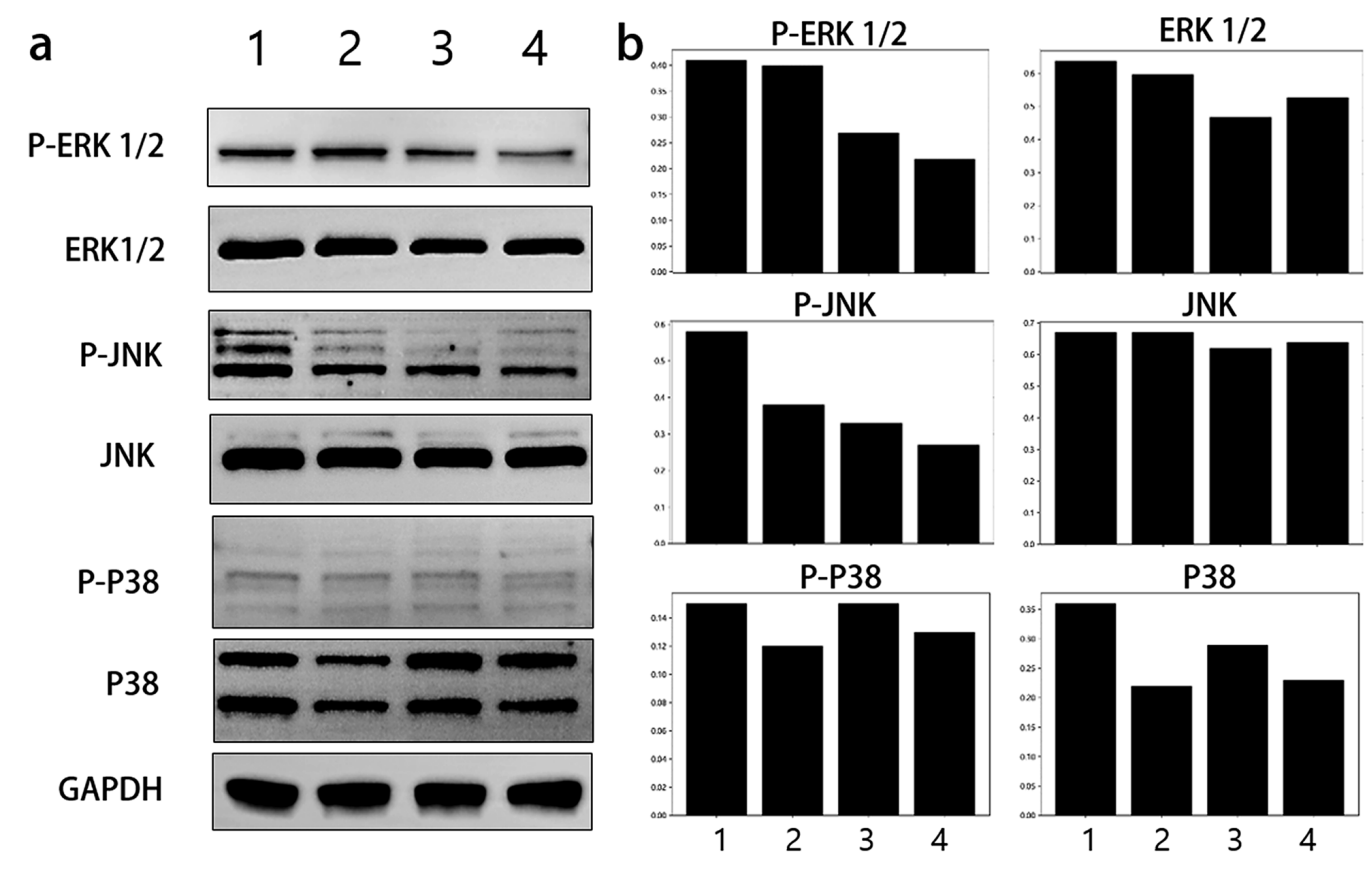
Effects of BDEs@lipo-SM on the phosphorylation of ERK1/2, JNK, and p38. Western blot analyses showed activation of the MAPK/ERK 1/2 pathway by BDEs@lipo-SM in Huh7 cells. (1: NC; 2: BDEs; 3:NAC; 4: BDEs@lipo-SM)

To further evaluate the mechanism of antihepatotoxic activity of BDEs@lipo-SM on APAP-induced hepatotoxicity, the protein expression of components of the mitogen-activated protein kinase (MAPK)/ERK 1/2 pathway was examined by western blotting. In a previous report, it was shown that the MAPK signaling pathway is involved in APAP-induced hepatotoxicity, but the regulatory mechanism of BDEs@lipo-SM on APAP-induced hepatotoxicity remains unknown, thus we analyzed protein expression of ERK1/2, JNK, and p38, which belong to the MAPK family. As shown in Fig. 5a, high levels of phosphorylation of ERK1/2, JNK, and p38 were observed in Huh7 cells. However, the level of protein expression of ERK1/2, JNK, and p38 was markedly suppressed following treatment with BDEs@lipo-SM. The relative band densities of ERK1/2, JNK, and p38 were also analyzed (Fig. 5b). It implies that the hybrid BDE strategy can be used to improve antihepatotoxicitic effects through the inhibition of the MAPK pathway and be a good candidate to prevent APAP-induced hepatotoxicity.

## Discussion

In recent years, PENs-based nanotechnologies have gained much attention because they exhibit a variety of functions ranging from cell-to-cell interaction and therapeutic applications. PENs are considered natural nanoparticles, with cargos of phospholipids, proteins, and genetic material that are not harmful. Unlike synthetic nanoparticles, PENs are safe for use as a topical and systemic treatment and may be suitable for certain therapeutic applications. PENs can be used as alternative drug delivery agents in a variety of medicinal applications because of their high stability, inherent biocompatibility, and specificity to target cells. Previous studies investigating PENs have reported the therapeutic potential of PENs and their cell signaling mechanism in various diseases.

Balloon flower root contains enriched bioactive compounds and isolated BDEs from their preparation include the various platycosides, specifically PD. A recent study showed that BDEs have great wound healing properties with anti-inflammatory and antioxidant effects in chronic skin wounds [29]. The enriched phytochemicals and bioactive molecules of BDEs can induce antioxidant activity against oxidative stress and can reduce the expression of pro-inflammatory cytokines. As shown in Fig. 1i, HPLC analysis indicates that BDEs contain different molecules, including PD, which plays a significant role in reducing oxidative stress and inflammation. Therefore, BDEs can be expected to contribute to counteract inflammatory disease and exert anti-apoptotic, and cell survival signaling. To achieve enhanced stability, greater structural integrity, and loading capacity of SM, we fabricated hybrid BDEs. Initially, we prepared SPC-based liposomes loaded with SM. Subsequently, the membranes of the BDEs and the SM-loaded liposomes were fused through sequential extrusion. To validate the stability of the fabricated BDEs@lipo-SM, size and zeta potential analyses were performed. The results showed an average particle size of 108.09 ± 14.26 nm and a zeta potential of -10.16 ± 0.58 mV. These values indicate that the engineered BDEs possess excellent stability in aqueous suspension.

Furthermore, to characterize the successful fusion of BDEs and liposomes, we used FRET and microscopy imaging (Figure S2). The results clearly showed that the fluorescence intensity of BDEs@lipo increased with the concentration of RhB-labeled BDEs, starting from the initial intensity of the donor C6 in liposomes, as observed in FRET analysis. Additionally, microscopy imaging revealed that the membranes of all BDEs were overlaid with the membranes of liposomes, indicating successful incorporation of BDEs and liposomes. This demonstrated that the BDEs-based drug delivery system can serve as an effective platform for therapeutic chemical drug delivery with excellent biocompatibility, no systemic immune response, and toxicity. We also showed that BDEs are superior to commercially available delivery systems, offering enhanced drug encapsulation efficiency, stability, long-term drug release/accumulation, and targeted delivery in vivo (Figures S3 and S4).

As proof of concept, using SM carried by BDEs-based drug delivery platform fused with liposomes as an example, BDEs@lipo treatment significantly enhanced anti-hepatotoxicity and anti-inflammatory effect in APAP-induced hepatotoxicity. BDEs@lipo-SM exhibit an advantage over original BDEs as they are rapidly captured by cells within 1 h and induce an increase in inhibitory activity against APAP-induced hepatotoxicity due to the synergistic effect of BDEs with SM. BDEs@lipo-SM distinctly evoke an anti-inflammatory immune response in immune cells due to their intrinsic bioactive components in BDEs, while reducing pro-inflammatory signals, albeit with no toxicity. Synergistically, BDEs@lipo-SM significantly increased the levels of GSH following liver injury, indicating that SM is successfully delivered in the liver through hybrid BDEs as a drug carrier and enhances cysteine availability, leading to an increase in GSH levels [30]. Synergistic BDEs@lipo-SM activity can more effectively inhibit the MAPK pathway and lead to liver cell recovery.

As described above, BDEs are obtained in high yield with high-grade purity and are expected to become reliable therapeutic carriers for the treatment of drug-induced hepatotoxicity. In the fabrication of the hybrid BDEs@lipo-SM, the BDEs remain structurally stable and retain their biological function, which means that BDEs@lipo-SM can act as a biocompatible systematic delivery system. To exploit the opportunities and potential applications for using BDEs as a drug delivery vehicle, more comprehensive applications based on the peculiar properties of BDEs and hybrid BDEs are necessary. Additionally, to expand the potential applications of BDEs, basic studies of hybrid PENs need to include a comprehensive discussion of the potential of current technology to develop efficient cargo loading, gene loading, and membrane engineering techniques to enhance specific delivery and avoid the systemic adverse effects induced by hybrid PENs over prolonged treatment.

## Conclusion

In this study, the incorporation of BDEs and liposome-loaded SM was significantly enhanced in vitro and in vivo, and BDEs demonstrated therapeutic potential for hepatoprotection and as an anti-inflammatory treatment, supported by evidence of reduced liver injury on histopathological examination compared to treatment with either non-modified BDEs and NAC alone. By inhibiting the MAPK pathway, BDEs@lipo-SM effectively inhibited APAP-induced hepatotoxicity by reducing the protein expression of ERK1/2, JNK, and p38. The results indicate that BDEs@lipo-SM could be successfully delivered in the liver and induce the required therapeutic effect, such as anti-inflammatory and promotion of immunity, due to the stability of BDEs@lipo-SM in vivo, as a carrier of intrinsic bioactive molecules and chemical agents. These findings provide the basis for further development and the fabrication of hybrid BDEs (or PENs) and applications as drug delivery agents to inhibit APAP-induced hepatotoxicity and liver injury. However, thus far, we have a limited understanding of the potential of PENS, and there are still several unanswered questions that need to be addressed, such as processes for highly optimized fabrication of hybrid BDEs and their systemic effects as vehicles of therapeutic agents in clinical trials for various diseases. Further research is required to improve the applicability of the hybrid BDEs as a drug delivery system.

### Electronic supplementary material

Below is the link to the electronic supplementary material.

Supplementary Material 1

Supplementary Material 2

## Data Availability

No datasets were generated or analysed during the current study.
