# Supplementary material for: A novel approach to alleviate acetaminophen-induced hepatotoxicity with hybrid balloon flower root-derived exosome-like nanoparticles (BDEs) with silymarin via inhibition of hepatocyte MAPK pathway and apoptosis
Source: Cell Commun Signal. 2024 Jun 18;22:334. doi: 10.1186/s12964-024-01700-z (PMC11184736; doi:10.1186/s12964-024-01700-z)
Supplement: Supplementary file 2 — Supplementary Material 2 [file 12964_2024_1700_MOESM2_ESM.docx]

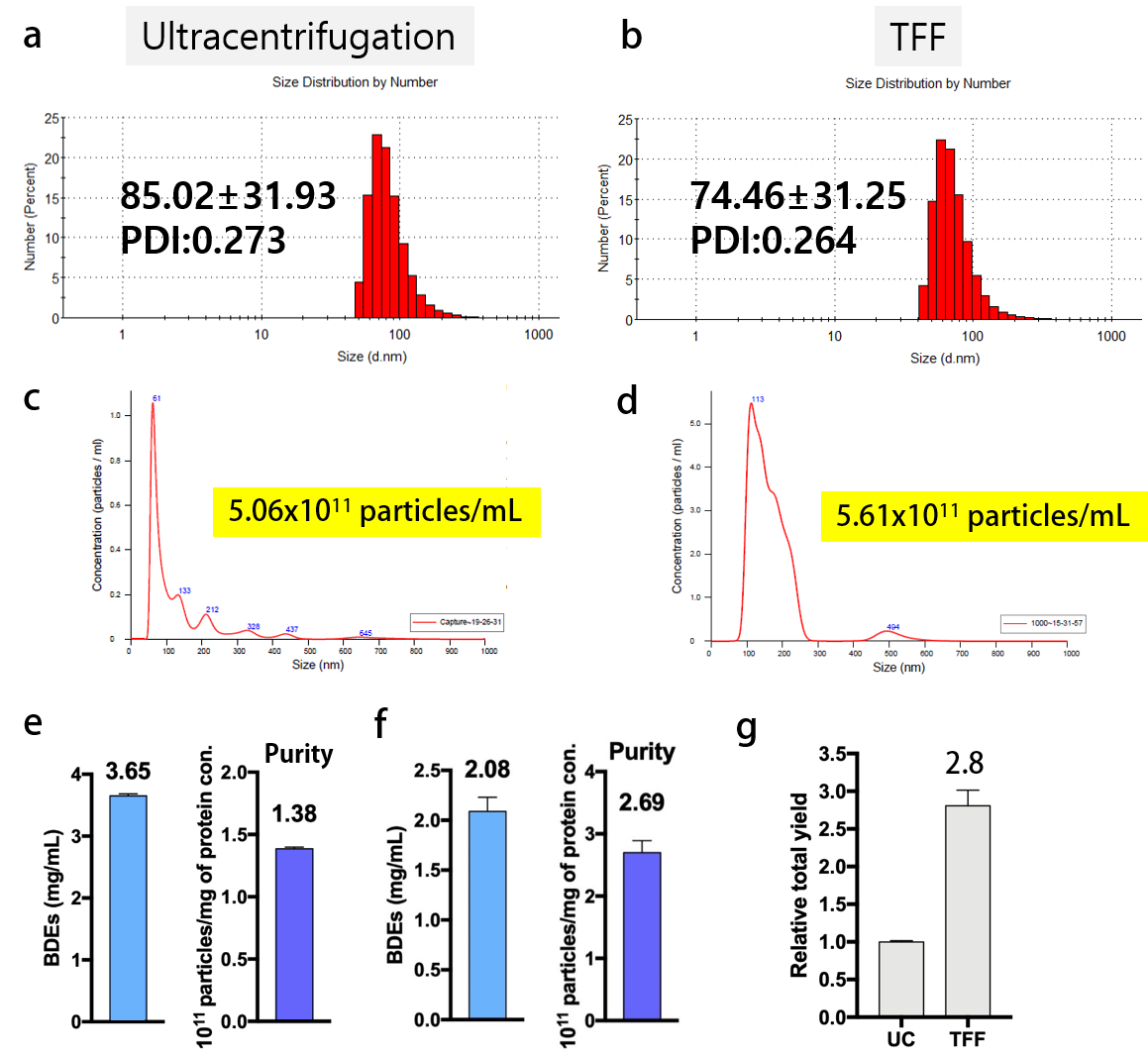


**Supporting information 1. Comparison of BDEs isolated by ultracentrifugation (UC) and TFF method.** (a, b) Size distribution of BDEs isolated by UC and TFF method, respectively. (c, d) The concentration of 5.06×10^11^ and 5.61×10^11^ particles/mL of BDEs isolated by two different methods was measured by nanoparticle tracking analysis (NTA), respectively. (e, f) The total protein concentration and the purity of BDEs isolated by UC and TFF method were calculated in the graph, respectively. (g) Relative total yield of BDEs isolated by UC and TFF method. The relative total yield of BDEs was achieved by multiplying the final volume of BDEs from each method by protein concentration in those samples. The relative yield ratio of BDEs isolated by TFF method was 2.8 folds higher than those isolated by UC.


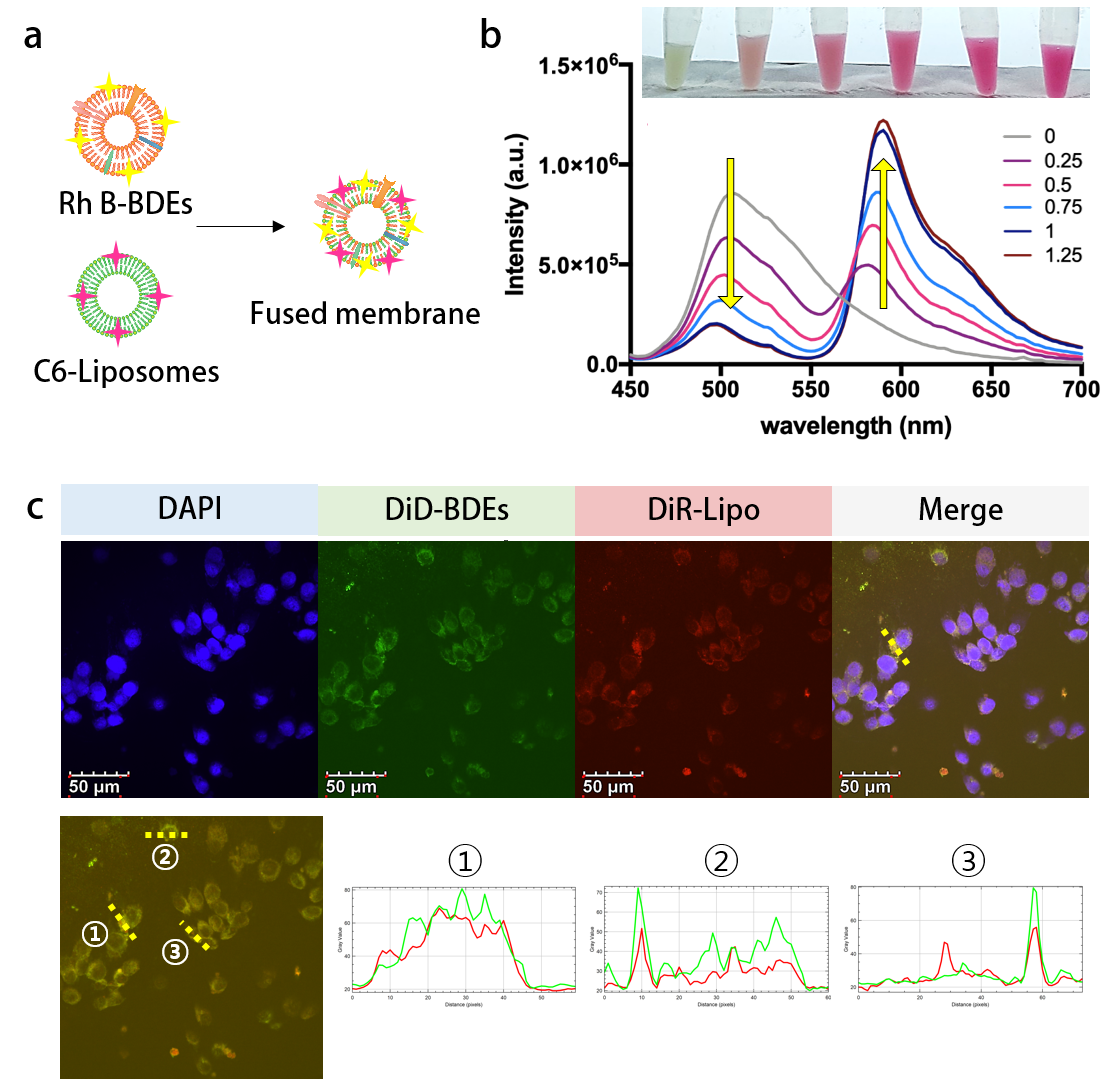


**Supporting information 2. FRET intensity of BDEs@lipo.** (a-b) The fluorescence spectra of BDEs@lipo when different amounts of RhB-labeled BDEs were input. C6-stained lipid membranes and RhB-labeled membranes were fused to form BDEs@lipo. (c) Representative super-resolution fluorescence images showed the colocalization of DiD-labeled BDEs and DiR-labeled liposomes in RAW 264.7 cells.


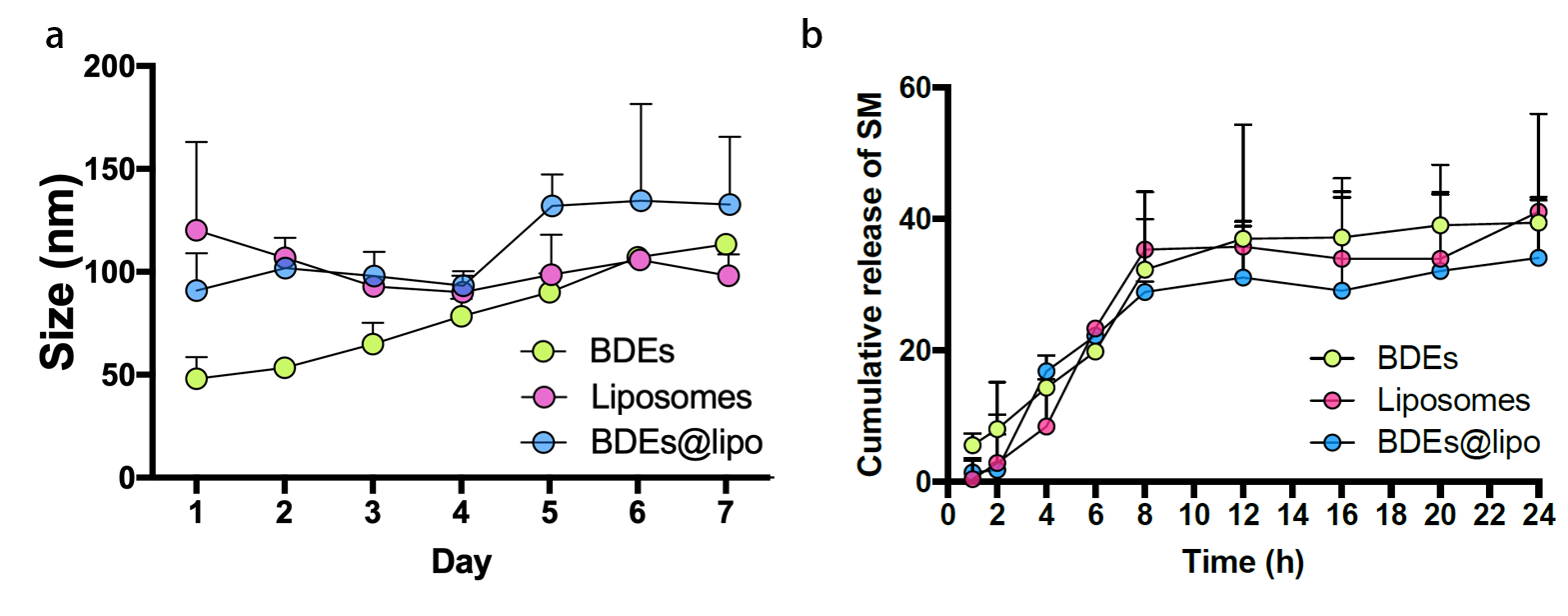


**Supporting information 3. Stability of BDEs, liposomes, and BDEs@lipo and *In vitro* drug release profile of SM loaded in BDEs, liposomes, and BDEs@lipo. Values are mean ±SD (n= 3).** (a) Size distribution of BDEs, liposomes, and BDEs@lipo for 7 days. (b) *In vitro* drug release of BDEs@lipo by the membrane dialysis method.

**
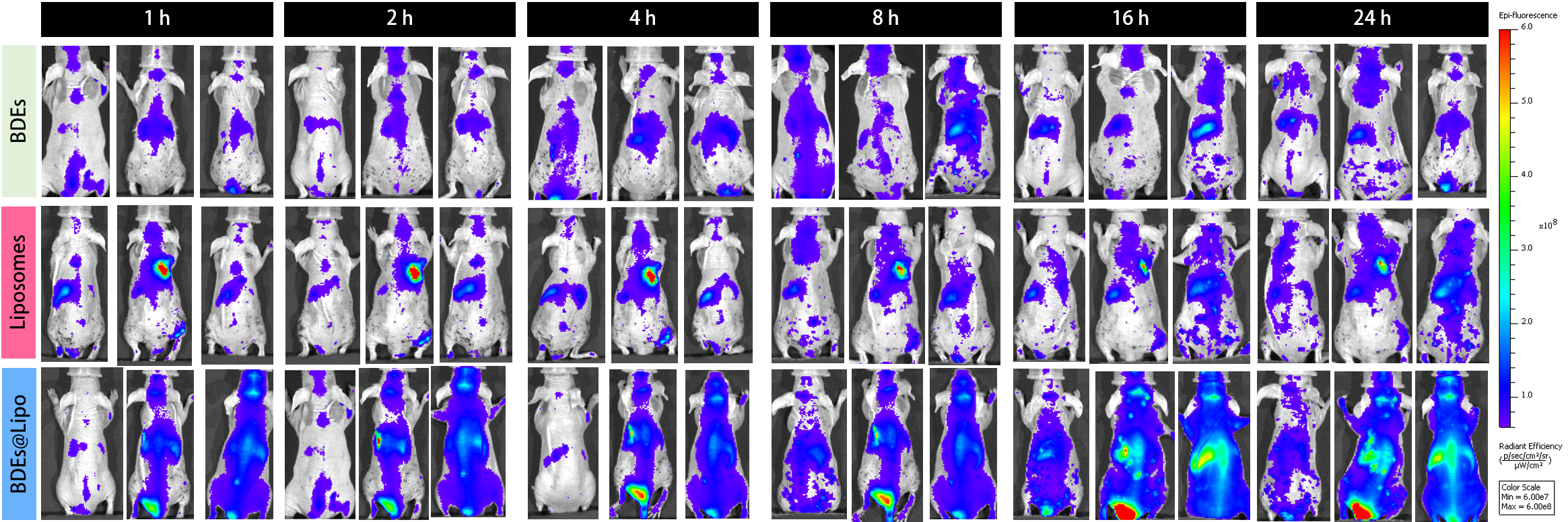
Supporting information 4. *In vivo* stability and distribution of BDEs, liposomes, BDEs@lipo.** In the presence of DiD-labeled BDEs, liposomes and BDEs@lipo, *in vivo* fluorescence imaging was performed in nude mice at different time points within 24 h post injection.
